# Manganese and Nickel Acetylacetonates as Curatives for Chloroprene Rubber Based on Heck’s Reaction

**DOI:** 10.3390/ma14040807

**Published:** 2021-02-08

**Authors:** Anna Dziemidkiewicz, Magdalena Maciejewska

**Affiliations:** Institute of Polymer and Dye Technology, Lodz University of Technology, Stefanowskiego Street 12/16, 90-924 Lodz, Poland; magdalena.maciejewska@p.lodz.pl

**Keywords:** crosslinking, chloroprene rubber, Heck reaction, metal acetylacetonates, mechanical properties

## Abstract

The commonly used curing system for chloroprene rubber (CR) is a combination of two metal oxides, such as magnesium oxide (MgO) and zinc oxide (ZnO). Application of MgO and ZnO enables to obtain a good balance between processability of rubber compounds and mechanical properties of the vulcanizates. Despite high activity in crosslinking reactions, ZnO is classified as ecotoxic to aquatic organisms, thus environmental legislation requires its quantity in technology to be limited. In our studies more environmentally friendly curing systems were applied, which enabled eliminating ZnO from CR compounds. These curing systems consisted of manganese acetylacetonate (Mn(acac)) or nickel acetylacetonate (Ni(acac)) and triethanolamine (TEOA) used as a base necessary to perform Heck’s reaction. Both metal acetylacetonates exhibited high activity in crosslinking reactions, which was confirmed by a great torque increment during rheometric measurements and high degree of elastomer crosslinking. The type of metal acetylacetonate and the amount of TEOA seemed to have less influence on the efficiency of the curing system than the filler used. Rubber compounds filled with carbon black (CB) were characterized by definitely shorter optimal vulcanization times and higher degree of crosslinking compared to CR composites filled with nanosized SiO_2_. Moreover, application of the proposed curing systems allowed to obtain CR vulcanizates with mechanical properties comparable with the benchmarks cured with metal oxides.

## 1. Introduction

Properties of chloroprene rubber (CR) are highly related to the changes in its molecular structure. The standard CR is characterized by highly regular structure and mainly consists of trans-units, which results in a high degree of polymer crystallinity [1]. The tendency of CR to crystallize under stress results in the high mechanical strength of the vulcanizates, for which CR is well known [2,3]. Consequently, unfilled CR vulcanizates reveal better mechanical properties than other elastomers (except for synthetic polyisoprene and natural rubber) arising from strain induced crystallization [3,4]. Furthermore, presence of the chlorine atom in the structure of CR deactivates the double bond and thus, the polymer is less liable to oxygen and ozone attack. Its widespread commercial use is, in part, due to its excellent weather resistance when compared to other rubbers under similar conditions [5,6,7]. Moreover, according to the increased polarity of CR in comparison with other (i.e., hydrocarbon) rubbers, CR vulcanizates are sufficiently resistant to mineral oils [4,8,9], gasoline, and some aromatic or halogenated solvents [8]. In contrast to the majority of other types of rubber, CR also exhibits a surprisingly greater level of resistance to microorganisms, such as fungi and bacteria [1,8]. Finally, owing to the presence of halogen in the rubber molecule, CR is more resistant to burning than exclusively hydrocarbon rubbers [1,3]. Therefore, it should be not surprising that, since its introduction to the marketplace, CR has become more than a simple oil-resistant replacement for natural rubber [6,8]. In comparison to other elastomers, CR is not characterized by a single outstanding property, but provides balance between some general properties [9]. Although these properties are not as good as those of some specialized rubbers, combined with relatively low price of CR, make this rubber a popular option for a broad range of applications [7,8].

To obtain a final product with preferred properties, it is necessary to crosslink CR before use. Vulcanization of CR differs from that of other diene rubbers, because electronegative chlorine disturbs both the double bond and the α-methylenic group making them inactive [9,10,11]. Chlorine atom retards the reaction of the electrophilic substitution common in the crosslinking mechanism of other unsaturated polymers [12,13]. Thus, sulfur vulcanization is ineffective and/or slow for the CR compounds [9,10,11]. To achieve a sufficient state of crosslinking, mostly used CR compounding formulations contain metal oxides, predominantly zinc oxide (ZnO) and magnesium oxide (MgO) [1,9,10].

Although CR may be crosslinked by ZnO, the use of ZnO alone usually results in a short scorch time and thus, relatively poor processing safety. On the other hand, in the absence of ZnO, the rheometer plot is relatively flat due to the low crosslinking degree of the elastomer. In turn, the rubber compounds with only MgO need rather long vulcanization times, while the crosslink density and mechanical properties of the vulcanizates are only modest [1,9,11]. Combination of MgO and ZnO provides a good balance between processability of rubber compounds and mechanical properties of the vulcanizates [8,9]. In this curing system, ZnO is the vulcanizing agent, while the role of MgO is to retard the crosslinking process and thus, extend the scorch time [12,14,15]. Practically used and commonly selected as a curing system for CR compounds is a combination of MgO (4 phr) and ZnO (5 phr), generally in the presence of ethylene thiourea (ETU) [1,9,11,12,14]. Application of this curing system results in the creation of both C–C and C–S bonds [12], leading to a noteworthy enhancement in the mechanical properties of the vulcanizates [16].

It should be noted that despite good performance, according to CMR (carcinogenic, mutagenic, reprotoxic) risk assessment: Repr. Cat. 2, R61—“May cause harm to the unborn child” [17], ETU is considered as toxic for reproduction. Thus, it is rational to expect that the use of ETU will be restricted or subject to authorization and replaced by safer accelerators [16]. Another problem is the possible release of zinc during production or service of rubber products, which may generate an excess of this element in the environment. Even though zinc is known as one of the least harmful of the heavy metals, above a critical content it becomes toxic, especially for the aquatic environment [18]. Thus, ZnO is classified as “very toxic to aquatic organisms and may cause long-term adverse effect in the aquatic environment” [17]. The precaution recommended in European Union Regulation is to avoid release to the environment. Hence, environmental EU legislation requires to decrease the use of ZnO and Zn-containing compounds in rubber products [19,20]. The increased emphasis on environment protection gives rise to a demand for products that entail the least impact on environmental pollution [19].

One of the closest studies to providing a substitution for ETU is that by Fuchs et al. [21]. The proposed 3-methyl-thiazolidint-thione-2 (MTT) was a promising solution resulting in higher scorch safety and higher vulcanization rate, which gave the opportunity of shorter vulcanization cycles. However, MTT happened to be dependent on the activity of ZnO (whereas ETU is not), and the health risk assessment has not been conducted yet. Nevertheless, MTT has gained technical importance [1]. Another successful study was performed by Berry [7] and allowed to develop alternative accelerators for CR vulcanization, which were found to be safer than ETU using computational analytical techniques to predict their toxicity levels. On the other hand, as candidates to commercialization, these accelerators still must undergo experimental toxicity studies, because the computational techniques are only appropriate for substances used in small quantities and are just used only as a guide to predict the toxicity of potential candidates. Another interesting approach was reported by Smejda-Krzewicka et al., who proved that CR may be effectively crosslinked with other metal oxides, such as tin oxide (SnO) [22], iron oxides (Fe_2_O_3_, Fe_3_O_4_) [23], or copper oxides (Cu_2_O, CuO) [24]. All presented examples enabled the elimination of both ETU and ZnO by using lower content of the proposed metal oxides compared to the reference curing system containing 5 phr of ZnO and 4 phr of MgO. An interesting review about new trends in reduction of zinc concentration methods in the vulcanization process was presented by S. Mostoni et al. [18]. Authors provided an overview of the main paths for designing highly efficient and innovative Zn-based materials to replace conventional ZnO in crosslinking activation. Proposed methods were based on the application of more active and well-dispersed zinc centers, which resulted with higher reactivity and accessibility towards the curing reagents and thus lower content needed.

In our previous studies [25,26,27,28], we reported the activity of metal acetylacetonates with different transition metals in the presence of triethanolamine (TEOA) as curing agents for CR. The crosslinking of CR with the proposed curing systems was proven to proceed in accordance with the mechanism of Heck reaction [26]. Application of these novel crosslinking systems allowed for the elimination of harmful vulcanization activators and accelerators, such as ETU and ZnO, making the CR compounds more environmentally friendly. Furthermore, high activity of metal acetylacetonate Me(acac)/TEOA systems, which was obtained with a very small amount of the crosslinking agent is also beneficial from an economical point of view, according to the relatively low price of TEOA and Me(acac) connected with small loadings needed.

Heck reaction has become one of the most powerful and versatile tools in the carbon-carbon bonds formation in organic synthesis [29,30,31]. It has been used for the synthesis of several intermediates in pharmaceuticals, antioxidants, UV absorbers, and fine chemicals [29,30]. However, despite being used in many applications in organic synthesis, the Heck reaction has not been utilized in the rubber industry so far.

**Scheme 1:**
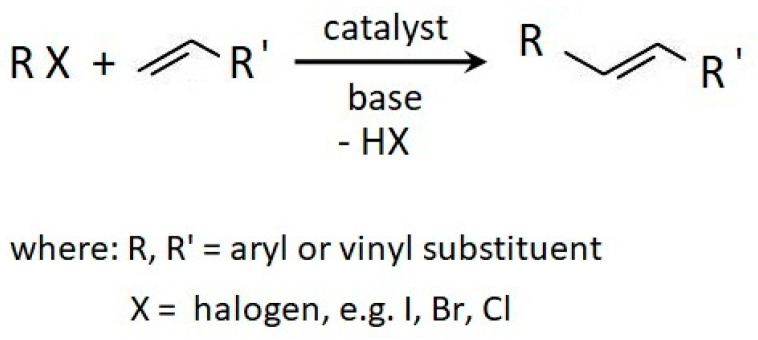
Heck coupling reaction of olefins with aryl or vinyl halides in the presence of catalyst and base [31,32].

Generally, Heck reaction is palladium-catalyzed coupling of olefins with aryl and vinyl halides, which proceeds in the presence of a base (Scheme 1) [31,32].

Organic and/or inorganic bases are necessary for regeneration of the catalyst by elimination of hydrogen halide (HX) in the final stage of the catalytic cycle and leaving the active Pd complex [29]. The essential part of the reaction is catalyst. A variety of metals along with a huge range of ligands were investigated as potential catalysts of Heck reaction [33]. However, due to the sensitivity of ligands to air and moisture, the high prices, toxicity, and non-recoverability of the proposed catalysts [31,34], simple palladium compounds, such as PdCl_2_ or Pd(OAc)_2_, have been progressively applied for the Heck reaction [34]. On the other hand, despite excellent performances of Pd catalysts, using low-cost transition metals (TMs) is a promising solution from an economic point of view [35]. Many positive results were achieved for some low cost transition metals, like Ni, Mn, Co, Cu, and Fe, which were reported to be active in Heck reactions [30,35,36,37].

In our previous studies, as an alternative to expensive Pd or Pt catalysts, much cheaper Fe, Ni, Mn, Cu, or Co acetylacetonates were applied. In addition, triethanolamine was successfully utilized to ensure the basic environment of the reaction. As was mentioned, the base is necessary to regenerate the catalyst by the bonding HCl produced in Heck reaction. CR was used to ensure unsaturation of the bonds and halogenation, which are obligatory in the reaction. From metal acetylacetonates tested, the highest activity in the vulcanization was exhibited by iron acetylacetonate (Fe(acac)), which was confirmed by the highest torque increment, the shortest optimal vulcanization time, and the greatest crosslinking degree of the elastomer. However, the high activity of Fe(acac) resulted in the short scorch time, which deteriorated the safety of rubber compounds processing. Therefore, in this work we focused on the application of manganese acetylacetonate (Mn(acac)) and nickel acetylacetonate (Ni(acac)) instead of Fe(acac). Moreover, the influence of different loadings of TEOA on the activity of the proposed curing systems was investigated.

## 2. Materials and Methods

### 2.1. Materials

Manganese (II) acetylacetonate (Mn(acac)) and nickel (II) acetylacetonate (Ni(acac)) (Sigma Aldrich, Poznań, Poland) in the presence of triethanolamine (TEOA) provided by Chempur (Piekary Śląskie, Poland) were used as catalysts of the crosslinking via Heck reaction. TEOA was applied to ensure an alkaline environment for the crosslinking reaction. Chloroprene rubber modified with xantogen disulfide (CR; XD grades, “Baypren” provided by LANXESS, Cologne, Germany) was applied as a rubber matrix. Fumed silica with specific surface area of 350–410 m^2^/g, particle size of 5–50 nm, and pH 3.7–4.5 (SiO_2_, Aerosil 380, Degussa A.G, Essen, Germany) and carbon black with specific surface area of 35–45 m^2^/g, particle size of 40–48 nm [38], and pH 7–10 (CB; N-550 supplied from Konimpex, Konin, Poland) were used as fillers.

### 2.2. Methods

The mixing procedure of CR compounds was maintained using a laboratory two-roll mill (Bridge, Sheffield, UK) (D = 200 mm and L = 450 mm) carried out with a friction of 1.15 at 30 °C. Applied formulations of rubber compounds are presented in Table 1. At the beginning, after 4 min of raw CR plasticizing, the filler was incorporated. In the next step, the crosslinking system was added and mixed another 6 min with the rubber compound. The total mixing time was around 10 min. All the compositions in Table 1 are presented as parts per hundred of rubber (phr). Rheometric measurements, which allowed to determine the optimal vulcanization time, were performed at 160 °C using Mon-Tech (Buchen, Germany) D-RPA 3000 rheometer. The vulcanization of rubber compounds was carried out at 160 °C using a hydraulic press with electrical heating.

After vulcanization of rubber compounds, the swelling procedure in toluene or toluene in chloroform vapor was conducted for the vulcanizates for 48 h in ambient temperature. Subsequently, when the weight of the swollen samples was measured, samples were dried in 50 °C for another 48 h. Then, the weight of the dried sample after swelling was measured. The degree of crosslinking (α_T)_) of the vulcanizates was calculated following the standard ISO 1817 [39], using Equation (1):

α_(T)_ = 1/Q_V_ (1)

where Q_V_ is the volume swelling determined following the Equation (2),

Q_V_ = Q_W_·q_r_/q_s_ (2)

where Q_W_ is the equilibrium swelling calculated with Equation (3), q_r_ is the rubber density (g/cm^3^), q_s_ is the solvent density (g/cm^3^).

Q_W_ = (m_sw_ − m_d_)/m_d_ (3)

where m_sw_ is the weight of the swollen sample (mg) and m_d_ is the weight of the dried sample after swelling (mg).

The tensile properties of the vulcanizates after and before aging were determined for dumbbell-shaped samples according to ISO-37 [40]. The tensile tests were carried out using a Zwick 1435 universal testing machine (Zwick Roell, Ulm, Germany) with a crosshead of 500 mm/min. According to the standard PN-ISO 188 [41], to investigate the resistance of CR to thermo-oxidative aging, vulcanizates were subjected to circulating air at a temperature of 70 °C for 7 days. Before and after the aging procedure, the tensile strength (TS) and the elongation at break (EB) were examined. The aging factor (A_f_) was determined from the following relationship [42]:

A_f_ = (TS · EB)_after aging_/(TS · EB)_before aging_ (4)

The hardness of the disc-shaped vulcanizates was investigated before and after the aging procedure. Measurements were carried out using a hardness tester Zwick Roell 3105 (Ulm, Germany) applying Shore’s method and following the PN-ISO 868 [43] standard.

## 3. Results and Discussion

### 3.1. Cure Characteristic of CR Compounds and Crosslinking Degree of the Vulcanizates

**Figure 1:**
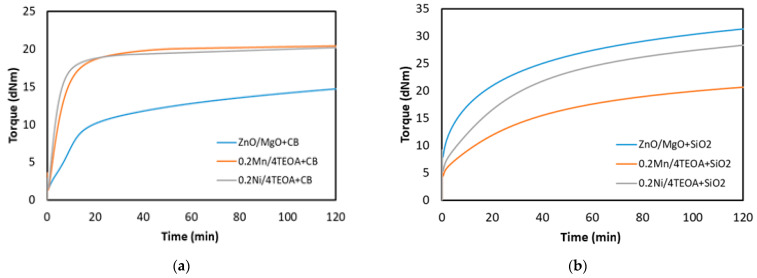
Rheometric curves at 160 °C for the CR compounds: (**a**) filled with carbon black (CB); (**b**) filled with SiO_2_.

**Figure 2:**
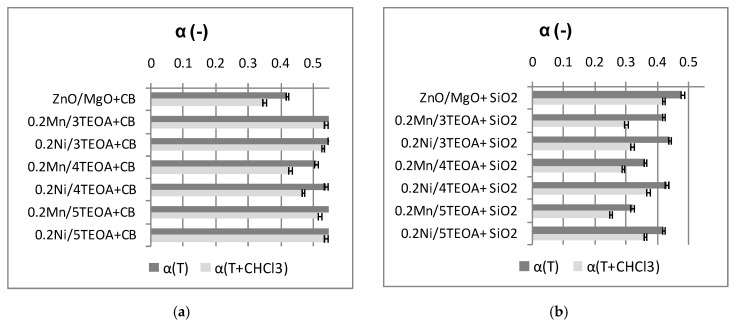
The degree of crosslinking calculated based on the solvent swelling measurements in toluene or toluene in chloroform vapor: (**a**) CB-filled vulcanizates; (**b**) SiO_2_-filled vulcanizates.

Cure characteristics of CR compounds (Figure 1 and Table 2), and the crosslinking degree of CR vulcanizates calculated based on the solvent swelling measurements in toluene or toluene in chloroform vapor (Figure 2), were evaluated to study the efficiency of the proposed curing systems containing manganese and nickel acetylacetonates (Mn(acac) and Ni(acac), respectively) on the crosslinking of CR compounds filled with carbon black (CB) or silica (SiO_2_).

Rheometric curves presented in Figure 1 indicate significant influence of the filler on the progress of crosslinking. Considering CB-filled rubber compounds with Me(acac) curing system, a sudden increase in torque can be observed at the very beginning of the plot. The plateau has already been reached in the first hour of the measurements, which resulted in definitely shorter optimal vulcanization time compared to reference CR compound cured with metal oxides. Unfortunately, it also resulted in shorter scorch time, which reduced the safety of the processing. In the case of SiO_2_-filled CR compounds, plots of cure were characterized by marching modulus, which explains longer optimal vulcanization times of all SiO_2_-filled rubber compounds compared to CB-containing rubber.

The first parameter presented in Table 2 is the minimum rheometric torque (M_L_), which correlates well with the Mooney viscosity of rubber compound at the temperature of measurement [2]. According to data presented in Table 2, the type of Me(acac) used as well as the content of TEOA did not significantly affect the viscosity of the uncured CR compounds filled with CB. It results from the very low content of those ingredients. However, the highest M_L_ values (2 dNm) were observed for rubber compounds with the lowest TEOA loadings. This may result from the reduction of the rubber compounds viscosity, when higher than 3 phr content of TEOA was applied. Considering the influence of the type of curing system used, CB-filled compounds with Me(acac) were characterized by M_L_ values in the range of 1.3–2 dNm, whereas for the reference sample containing ZnO/MgO system this value was 1.4 dNm. In the case of SiO_2_-filled rubber compounds, the M_L_ were within the range of 4.4–6.7 dNm, whereas for the benchmark M_L_ was approximately 7.3 dNm. Regarding SiO_2_-filled CR, the curing systems containing Me(acac) and TEOA had more pronounced effect on the M_L_, especially when 4 or 5 phr of TEOA was applied. M_L_ values were reduced by 2–3 dNm compared to the benchmark containing ZnO/MgO system. Considerable influence of the filler used on the M_L_, so the viscosity of uncured rubber compounds, was observed. It should be noticed that nanosized silica increases the viscosity of rubber compounds and their stiffness much more than CB (significantly higher M_L_ compared to CB-filled rubber), therefore the softening effect of higher content of TEOA was more visible in the SiO_2_-filled rubber compounds. Lower M_L_ resulting from the lower viscosity of the rubber compounds, is preferred from a technological point of view due to their easier processing.

The cure characteristics, so the progress of crosslinking, may be monitored by the measurements of development of the torque as a function of time at a given temperature [2,44]. The torque increment (ΔM) during crosslinking is commonly used as an indirect indication of the crosslinking state of the elastomer [45]. Considering activity of Mn(acac) and Ni(acac) as crosslinking agents for CR compounds, no significant differences were achieved in ΔM values considering the measurement error. Although still moderately, the influence of TEOA on the activity of the proposed curing systems depended on the type of filler used. For CB-filled rubber compounds, the activity of metal acetylacetonates increased slightly with the higher content of TEOA, and thus the greatest ΔM was exhibited by CR compounds with 5 phr of TEOA. A more surprising effect of the curing system’s composition was observed for SiO_2_-filled rubber compounds. Despite the lack of clear relations between the amount of TEOA and the crosslinking efficiency, the greatest ΔM values, for both Mn(acac) and Ni(acac), were observed for rubber compounds with the lowest content of TEOA. It may be due to the polar nature of SiO_2_ and partial adsorption of the Me(acac) on its surface. In this case, higher loadings of TEOA, which is a surfactant, not only does not improve the activity of Me(acac), but also may decrease the friction between the rubber compound and rotor during the rheometric measurements due to the low viscosity of TEOA.

It is commonly known that the surface of silica, especially nanosized SiO_2_, shows a strong ability for curatives adsorption [46], especially for Aerosil 380 silica, for which the BET surface area value is not only connected with a particles size, but also certain degree of surface roughness. On the other hand, because of an acidic character of SiO_2_, adsorption of TEOA with the basic character also would be preferable [46]. The dependence of the efficiency of the crosslinking process and the activity of the crosslinking system on the surface area of the silica was also confirmed by Sowińska et al. [47]. The influence of the ionic liquids immobilized on the surface of solid supports with different specific surface areas on the vulcanization was investigated. As a solid support, different silicas, such as Ultrasil VN3 silica (165 m^2^/g) and silica nanopowder (560 m^2^/g), were used. According to presented results, vulcanizates containing silica nanopowder with greater specific surface area were characterized by lower crosslink density than those with Ultrasil VN3. Moreover, rubber compounds containing silica nanopowder did not cure at 100 °C opposite to those containing Ultrasil VN3.

Considering the activity of the curing systems tested, metal acetylacetonates exhibited higher activity for CB-filled rubber compounds compared to the benchmark containing ZnO/MgO, whereas for SiO_2_-filled CR compounds higher curing activity was observed for metal oxides. Application of Me(acac)/TEOA curing systems in CB-containing rubber compounds allowed to obtain ΔM in the range of 17.1–21.2 dNm, whereas for the benchmark with ZnO/MgO ΔM was only 14.1dNm. In the case of SiO_2_–filled rubber compounds, slightly lower activity of the Me(acac)/TEOA curing systems compared to the ZnO/MgO resulted in ΔM values in the range of 16.3–23.4 dNm, whereas for the reference rubber compound ΔM was approximately 24.3 dNm. Taking into account very low content (0.2 phr) of Me(acac) in the curing system, its partial adsorption on the surface of silica considerably reduces the efficiency of crosslinking reactions. On the other hand, the content of ZnO and MgO (5 phr and 4 phr, respectively) in the reference curing system is much higher than Me(acac) and these metal oxides are not adsorbed on the filler’s surface, which results in the higher activity of the ZnO/MgO system compared to Me(acac)/TEOA.

Another parameter associated with the efficiency of crosslinking is optimal vulcanization time (TC90). Shorter vulcanization time is technologically preferred, since shorter TC90 determines lower costs of the vulcanization process. Considering the standard deviation of presented results, the type of Me(acac) used and the content of TEOA did not significantly affect the TC90, but a great influence of the filler used was observed. CB-filled rubber compounds containing Me(acac) exhibited TC90 in the range of 21–27 min, whereas SiO_2_-containing CR composites were characterized by definitely longer TC90 (8999 min). Regardless of the filler used, benchmarks containing ZnO/MgO system exhibited similar TC90 (96 min for CB and 98 min for SiO_2_-containing CR). Most importantly, Me(acac)/TEOA systems reduced TC90 of CB-filled rubber compounds by approximately 70 min compared with the benchmark containing metal oxides. This is very beneficial for technological reasons. On the other hand, Me(acac)/TEOA systems did not significantly affect the TC90 of SiO_2_-filled rubber compounds. Only in the case of CR compounds containing 5 phr of TEOA, TC90 was approximately 7 min shorter than that of the reference composite cured with ZnO/MgO. This may be due to the previously mentioned adsorption of the Me(acac) on the surface of nanosized SiO_2_.

The last parameter determined from rheometric measurements was the scorch time (TS2) at 160 °C. Scorch safety, which is specified by the scorch time, is the period during which the compound may be maintained at an elevated temperature and still be characterized by plastic properties [2]. It must be long enough to allow mixing and shaping rubber compounds before the crosslink formation starts [2,14]. CR compounds exhibited the scorch times in the range of 0.4–0.8 min, regardless of the filler type and the curing system used (Me(acac)/TEOA or ZnO/MgO). The results obtained were within the range of experimental error. Thus, it was concluded that the proposed curing systems containing Mn(acac) or Ni(acac) did not significantly affect the scorch safety of CR compounds at 160 °C.

Another parameter related to the efficiency of the crosslinking process is the degree of crosslinking calculated on the basis of equilibrium swelling in toluene, which is presented in Figure 2. No significant influence of the type Me(acac) used or the content of TEOA on the crosslinking degree of CR vulcanizates was observed. On the other hand, the effect of the filler applied is evident. Me(acac)/TEOA systems exhibited higher activity in the crosslinking of CB-filled rubber compounds resulting in higher crosslinking degree of the vulcanizates compared to those containing SiO_2_. Vulcanizates containing CB were characterized by α_T_ values in the range of 0.51–0.60, when those containing SiO_2_ revealed α_T_ values in the range of 0.32–0.44. As mentioned before, lower efficiency of the proposed curing system in the presence of SiO_2_ may result from adsorption of the Me(acac) on the surface of SiO_2_, due to its polar character. When compare to the reference curing system, which contained ZnO and MgO, using Me(acac)/TEOA resulted in a slightly higher α_T_ of the CB-filled vulcanizates, whereas for SiO_2_-filled CR α_T_ were slightly lower than that of the benchmark. However, considering the experimental error, it could be concluded that Me(acac)/TEOA curing systems did not significantly affect the crosslinking degree of the CR elastomer compared to ZnO/MgO.

**Scheme 2:**
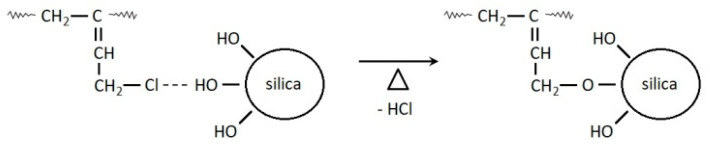
Reaction between the silanol groups of silica and the allylic chlorine atoms of CR proposed by Sae-oui et al. [45].

Furthermore, in Figure 2 the differences between the degree of elastomer crosslinking determined in toluene (α_T_) and toluene in chloroform vapor (α_T+CHCl3_) are shown. Those differences may indicate the presence of non-covalent network nodes (crosslinks) in the structure of the elastomer, which may improve the mechanical strength of the CR vulcanizates. More pronounced differences between α_T_ and α_T+CHCl3_ values were determined for the vulcanizates containing SiO_2_. It should be noted that for the SiO_2_–filled vulcanizates, these differences may result not only from the formation of non-covalent crosslinks in the elastomer network, but also from the interactions between precipitated silica and CR, i.e., formation of some weak bonds between CR and SiO_2_, which were destroyed in the presence of chloroform vapor. Choi [48] and Das et al. [49] proposed the interaction between the chlorine from CR and the hydrogen atom from the silanol group of silica through the hydrogen bonds. On the other hand, Sae-oui et al. [45] confirmed the probable mechanism of interactions between the silica surface and CR, which was previously proposed by Wang et al. [50]. According to this mechanism, at high temperature, the bond formation between reactive allylic chlorine atoms and the silanol groups of silica occurs with a release of HCl, as shown in Scheme 2 [45].

### 3.2. Mechanical Properties and Resistance to Thermo-Oxidative Aging of Vulcanizates

**Figure 3:**
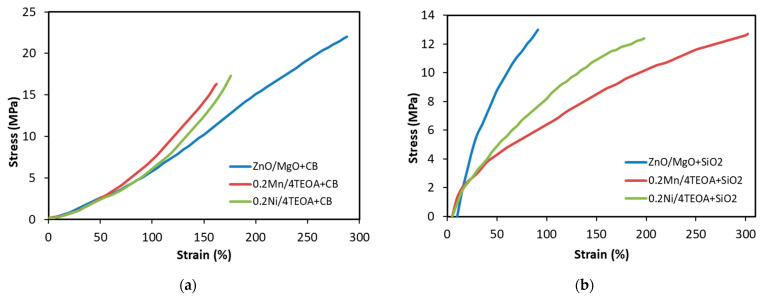
Stress-strain curves of CR vulcanizates: (**a**) filled with CB; (**b**) filled with SiO_2_.

Having investigated the activity of the proposed curing systems in the CR crosslinking, we then examined the effect of these curing systems on the mechanical properties and resistance to thermo-oxidative aging of the CR vulcanizates. Results are presented in Figure 3 and Table 3 and Table 4.

The filler and the curing system used had a significant influence on the stress-strain curves of CR vulcanizates, and so their tensile properties. Higher activity of the Me(acac)/TEOA curing systems in the crosslinking of CB-filled CR resulted in the higher tensile strength of the vulcanizates in comparison to SiO_2_-filled elastomers. Moreover, the high crosslinking degree of the CB-filled vulcanizates cured with Me(acac)/TEOA systems resulted in their worse mechanical properties (lower tensile strength (TS) and elongation at break (EB)) in comparison to vulcanizate cured with ZnO and MgO (Figure 3a). On the other hand, application of nanosized silica resulted in increased stiffness, when compared to CB-filled vulcanizates, which is especially visible at the very beginning of the stress-strain plots. However, lower activity of the Me(acac)/TEOA curing system in SiO_2_-filled CR resulted in higher EB of the vulcanizates compared to the benchmark cured with metal oxides (Figure 3b), but quite comparable TS.

Since tensile mechanical properties of the vulcanizates show a non-linear stress-strain nature, the modules at 50%, 100%, and 150% relative elongation of the vulcanizates were presented in Table 3. The SE_50_ of SiO_2_-filled vulcanizates were higher compared to those of CB-filled CR. It indicates higher stiffness of the vulcanizates filled with nanosized SiO_2_ compared to the vulcanizates containing CB. Analyzing the data given in Table 3, next presented parameter is SE_100_, which is related to the crosslinking degree of the vulcanizates and activity of the filler. The CB-filled vulcanizates exhibited SE_100_ in the range of 6.4–9.5 MPa, whereas for SiO_2_-filled vulcanizates the values of SE_100_ were slightly lower and ranged from 4.1 MPa to 8.8 MPa, that confirmed the higher activity of Me(acac)/TEOA system in the crosslinking of CB-filled CR. Moreover, CB-filled vulcanizates containing Me(acac) exhibited higher SE_100_ compared to the benchmark cured with ZnO/MgO. The highest SE_100_ was demonstrated by the vulcanizate with the highest α_T_. In the case of SiO_2_–filled CR, the reference vulcanizate cured with ZnO/MgO exhibited the elongation at break lower than 100%, thus it was impossible to compare the SE_100_ of the benchmark and vulcanizates containing Me(acac). CB-filled vulcanizates cured with the proposed curing systems exhibited the SE_150_ in the range of 12.4–15.8 MPa, so significantly higher than those of SiO_2_-filled CR (5.1–10.9 MPa). It was due to the lower crosslinking degree of the vulcanizates filled with nanosized SiO_2_, which probably resulted from the adsorption of the curatives on the silica surface, decreasing their activity in the crosslinking reactions.

Another parameter related to the degree of elastomer crosslinking is the elongation at break (EB). For CB-filled elastomer, the highest EB was shown by the reference vulcanizates, which was characterized by the lowest crosslinking degree. Considering the vulcanizates filled with CB and cured with Me(acac), the slight decrease in EB with the increase of the TEOA content was observed. It confirms that the efficiency of crosslinking increases with TEOA content. The same relationship was achieved for ΔM, which is an indirect measure of the elastomer crosslinking degree. Regarding the SiO_2_-filled vulcanizates, similar to ΔM and α_T_, the correlation between the type of Me(acac) used and the TEOA content was not so clear as for CB-filled CR. However, the lowest EB observed for the benchmark containing ZnO/MgO correlated well with the highest crosslinking degree of this elastomer, when the highest EB was determined for the vulcanizate with the lowest degree of crosslinking (0.2 Mn/5TEOA+SiO_2_).

As far as the tensile strength (TS) of the vulcanizates is concerned, higher curing activity of Me(acac)/TEOA systems in the CB-filled CR resulted in the higher TS compared to the SiO_2_-filled vulcanizates. CB-filled CR cured with Me(acac) exhibited TS in the range of 16.0–20.6 MPa, whereas SiO_2_-filled vulcanizates showed lower TS, in the range of 10.3–13.6 MPa. In the case of CB-filled elastomer, the highest TS was exhibited by the vulcanizates with the lowest TEOA content for both Mn(acac) and Ni(acac). Although the influence of TEOA amount on the TS was slight, it may indicate that higher amount of TEOA, which results in the higher degree of elastomer crosslinking, deteriorates mechanical properties of the CR vulcanizates. It is well known, that the crosslink density strongly influences the mechanical properties of the elastomer [44]. Elastic properties such as tensile and dynamic modulus, hardness, resilience, tear, and tensile strength enlarge with the increase of the crosslink density [2]. However, the mechanical strength of the vulcanizate increases with the number of crosslinks in the elastomer network only to some extent [44]. Further growth in the crosslink density results in the vulcanizates that tend toward brittle behavior and thus, such properties as hardness, tear, and tensile strength begin to decrease [2]. A high crosslink density limits chain motions and a highly crosslinked network is unable of dissipating much energy. Consequently, rather easy, brittle fracture at low elongation is observed [44]. For CB-filled vulcanizates, the greatest TS was observed for the benchmark cured with ZnO/MgO (22 MPa). Lower TS of the vulcanizates with Me(acac)/TEOA may result from the higher activity of these curing systems and the formation of C=C bonds [26] in their presence and thus “tighter” network comparing to reference elastomer cured with ZnO/MgO. In case of SiO_2_-filled CR, there was no clear correlation between the content of TEOA and TS of the vulcanizates. However, higher TEOA content resulted, to some extent, in better TS, because the highest TS (12.4–13.6 MPa) were observed for the vulcanizates with 4 phr of TEOA for both Me(acac) and for the vulcanizate with 0.2 phr of Ni(acac) and 5 phr of TEOA. It is worth noting that the last-mentioned vulcanizate exhibited TS comparable to the benchmark containing ZnO/MgO (13 MPa). It allows to conclude that application of optimal amount of Me(acac) and TEOA enables to obtain mechanical properties similar to the reference vulcanizate cured with metal oxides.

The last parameter presented in Table 4 is hardness (H), which usually rises with increasing the crosslink density of the vulcanizate [44]. No less important is the effect of the filler used. Analyzing the data given in Table 4, a significant influence of the filler type on the hardness of CR vulcanizates was observed. SiO_2_-filled vulcanizates were characterized by significantly higher H compared to CB-filled elastomers. This resulted from the use of nanosized silica, which significantly increased the stiffness of vulcanizates, and consequently their hardness. SiO_2_-filled vulcanizates cured with Me(acac) exhibited the hardness in the range of 71–79 ShA, whereas CB-filled vulcanizates demonstrated lower hardness (59–63 ShA). In the case of CB-filled CR composites, higher hardness was observed for the vulcanizates with higher TEOA loadings. Regardless of the filler used, the reference vulcanizates cured with ZnO/MgO showed higher hardness compared to the vulcanizates containing Me(acac)/TEOA system (68 ShA and 84 ShA for the vulcanizates with CB and SiO_2_, respectively).

The proposed curing systems contain transition metal acetylacetonates. Owing to the fact that transition metals can deteriorate the aging resistance of rubber products, thermo-oxidative aging studies were performed. The aging coefficient (A_f_) was determined based on the changes in the mechanical properties (TS and EB) of the vulcanizates due to the aging process. Results are presented in Table 4. A value of aging coefficient close to 1 represents a sample with perfect aging resistance. It indicates that the aging process had no effect on the TS and EB of the vulcanizate. Analyzing the values of A_f_, it was observed that the fillers seemed to have a significant effect on the resistance of CR to thermo-oxidative aging, regardless of the curing systems used. CB-filled vulcanizates exhibited better aging resistance than the SiO_2_-filled CR. Better aging resistance of the CB-filled elastomers probably resulted from the fact that CB may act as an effective radical trapping agent [51]. The reference vulcanizate filled with CB was characterized by the best aging resistance of all samples, since the A_f_ was approximately 1. Application of Me(acac)/TEOA curing systems deteriorated the aging resistance of CB-filled vulcanizates (A_f_ in the range of 0.5–0.8) compared to the benchmark cured with ZnO/MgO. Considering the SiO_2_–filled CR, Me(acac)/TEOA curing systems had no detrimental effect on the resistance to thermo-oxidative aging. The benchmark with ZnO/MgO was characterized by A_f_ 0.3, whereas for Me(acac)-containing vulcanizates, the A_f_ values were in the range of 0.2–0.6.

**Figure 4:**
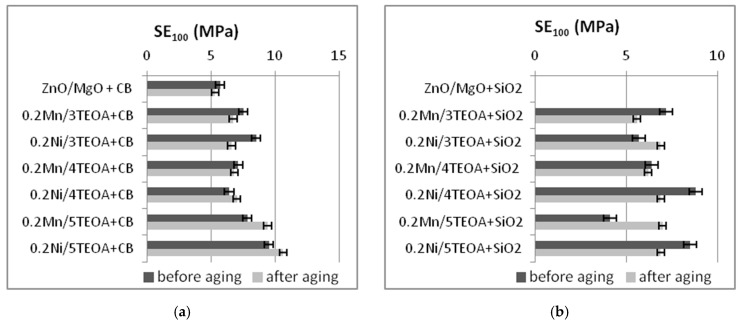
Influence of thermo-oxidative aging on the stress at 100% relative elongation (SE_100_) of the vulcanizates: (**a**) filled with CB; (**b**) filled with SiO_2_.

**Figure 5:**
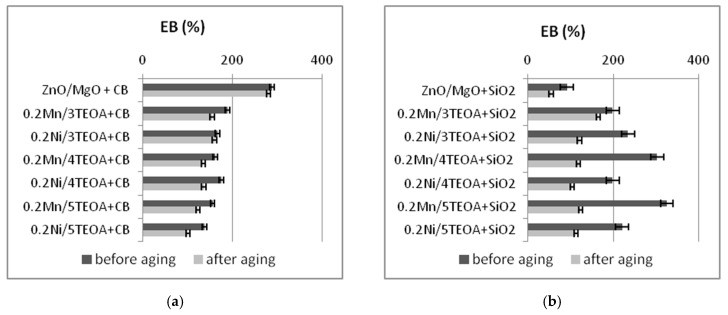
Influence of thermo-oxidative aging on the elongation at break (EB) of the vulcanizates: (**a**) filled with CB; (**b**) filled with SiO_2_.

**Figure 6:**
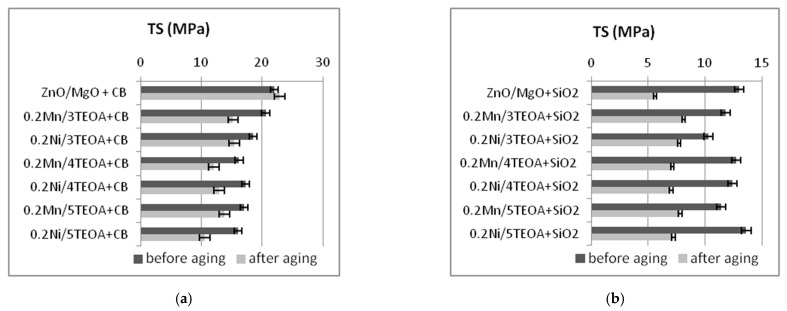
Influence of thermo-oxidative aging on the tensile strength (TS) of the vulcanizates: (**a**) filled with CB; (**b**) filled with SiO_2_.

**Figure 7:**
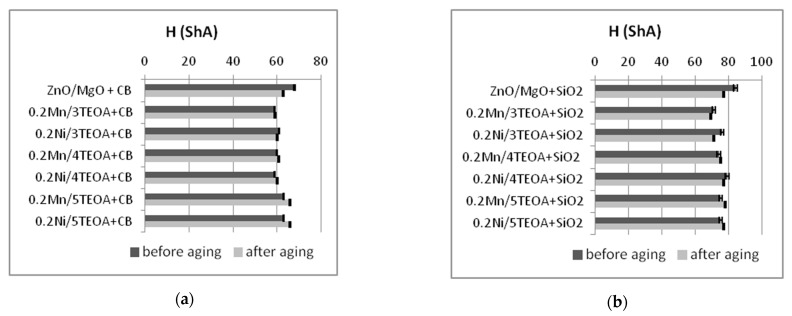
Influence of thermo-oxidative aging on the hardness (H) of the vulcanizates: (**a**) filled with CB; (**b**) filled with SiO_2_.

The effect of thermo-oxidative aging on the mechanical properties of CR vulcanizates, such as SE_100_, TS, EB, and H is presented in Figure 4, Figure 5, Figure 6 and Figure 7. Considering the differences in SE_100_ values before and after aging procedure (Figure 4) and taking into account the experimental error, no significant changes of SE_100_ were observed for most vulcanizates, especially those filled with CB. However, a more pronounced influence of thermo-oxidative aging on the SE_100_ was achieved for the vulcanizates containing SiO_2_, which exhibited worse resistance to thermo-oxidative aging compared to CB-containing CR.

The reduction in EB of the vulcanizates after aging process (Figure 5) may result from the increase in the elastomer crosslinking degree. Undeniably smaller changes in EB were observed for the vulcanizates containing CB compared to SiO_2_-filled elastomer. In case of SiO_2_-filled vulcanizates, definitely lower EB values after the aging procedure may also confirm some further crosslinking reaction between CR and SiO_2_. For both fillers, the lowest changes in EB were achieved for the reference vulcanizates containing ZnO/MgO.

The lower TS of the CR composites after thermo-oxidative aging (Figure 6) may indicate both degradation and crosslinking processes. It should be noticed that for the SiO_2_–filled vulcanizates, reduction in TS was definitely more pronounced compared to the CB-filled elastomers. Regardless of the filler used, the differences between the TS of non-aged and aged CR vulcanizates cured with Me(acac) were in the range of 2.6–6.4 MPa.

The effect of thermo-oxidative aging on the hardness of the vulcanizates is presented in Figure 7. As it was for A_f_, the influence of the curing system used on the aging resistance of the vulcanizates is evident. Considering ZnO/MgO-containing elastomers, thermo-oxidative aging reduced the hardness of the vulcanizates by 5–7 ShA compared to the non-aged samples. In the case of Me(acac)-cured rubber compounds, smaller changes in H values were observed regardless of the filler used (1–3 ShA for most of the vulcanizates).

## 4. Conclusions

Manganese (II) and nickel (II) acetylacetonates exhibited high activity in the crosslinking of CR compounds, which was confirmed by a great torque increment during rheometric measurements and the high degree of elastomer crosslinking. The type of metal acetylacetonate and the amount of triethanolamine seemed to have less influence on the efficiency of the curing system than the filler used. Greater activity of the proposed curing systems was observed for CB-filled CR compared to SiO_2_-containing rubber compounds. Considering the torque increment and the elastomer crosslinking degree, metal acetylacetonates exhibited higher activity in the CB-filled rubber compounds compared to the benchmark containing ZnO/MgO. On the other hand, for SiO_2_-filled CR compounds higher curing activity was observed for metal oxides. Most importantly, due to their high activity in rubber crosslinking, Me(acac)/TEOA systems reduced optimal vulcanization times of CB-filled rubber compounds by approximately 70 min compared to the benchmark containing metal oxides.

Higher curing activity of Me(acac)/TEOA systems in the CB-filled CR resulted in higher tensile strength in comparison with the SiO_2_-filled vulcanizates. In the case of CB-filled elastomer, the highest TS was exhibited by the vulcanizates with the lowest TEOA content for both Me(acac). Although the influence of TEOA amount on the TS was slight, it may indicate that higher amount of TEOA, which results in the higher degree of elastomer crosslinking, deteriorates mechanical properties of the CR vulcanizates. Regarding CB-filled vulcanizates, the greatest TS was observed for the benchmark cured with ZnO/MgO. In case of SiO_2_-filled CR, there was no clear correlation between the content of TEOA and TS of the vulcanizates. However, application of optimal amount of Me(acac) and TEOA enabled to obtain mechanical properties similar to the reference vulcanizate cured with metal oxides.

The filler type significantly affected the hardness of CR vulcanizates. SiO_2_-filled vulcanizates were characterized by significantly higher hardness compared to CB-filled elastomers. Regardless of the filler used, vulcanizates cured with ZnO/MgO showed higher hardness compared to the vulcanizates containing Me(acac)/TEOA system.

The type of filler had a significant effect on the resistance of CR vulcanizates to thermo-oxidative aging, regardless of the curing system used. Application of Me(acac)/TEOA curing systems deteriorated the aging resistance of CB-filled vulcanizates, whereas it had no detrimental effect on the resistance to thermo-oxidative aging of SiO_2_-containing CR in comparison with the benchmarks cured with metal oxides.

## Figures and Tables

**Table 1:** General compositions of the chloroprene rubber (CR)-based rubber compounds (parts per hundred of rubber; phr).

Compound Description	CR	CB	SiO_2_	TEOA	Me(acac)	ZnO/MgO
Rubber Compounds Filled with CB						
ZnO/MgO+CB	100	30	-	-	-	5ZnO+4MgO
0.2Mn/3TEOA+CB	100	30	-	3	0.2 Mn	
0.2Ni/3TEOA+CB	100	30	-	3	0.2 Ni	
0.2Mn/4TEOA+CB	100	30	-	4	0.2 Mn	
0.2Ni/4TEOA+CB	100	30	-	4	0.2 Ni	
0.2Mn/5TEOA+CB	100	30	-	5	0.2 Mn	
0.2Ni/5TEOA+CB	100	30	-	5	0.2 Ni	
Rubber Compounds Filled with SiO_2_						
ZnO/MgO+SiO_2_	100	-	30	-	-	5ZnO+4MgO
0.2Mn/3TEOA+SiO_2_	100	-	30	3	0.2 Mn	
0.2Ni/3TEOA+SiO_2_	100	-	30	3	0.2 Ni	
0.2Mn/4TEOA+SiO_2_	100	-	30	4	0.2 Mn	
0.2Ni/4TEOA+SiO_2_	100	-	30	4	0.2 Ni	
0.2Mn/5TEOA+SiO_2_	100	-	30	5	0.2 Mn	
0.2Ni/5TEOA +SiO_2_	100	-	30	5	0.2 Ni	

**Table 2:** Cure characteristics of CR compounds at 160 °C: ML—minimum torque, ∆M—torque increment, TS2—scorch time (time at 2% of ΔM), TC90—optimal vulcanization time (time at 90% of ΔM).

Rubber Compound	M_L_ (dNm)	ΔM (dNm)	TS2 (min)	TC90 (min)
Rubber Compounds Filled with CB				
ZnO/MgO+CB	1.4 ± 0.1	14.1 ± 1.7	0.8 ± 0.1	96 ± 2
0.2Mn/3TEOA+CB	2.0 ± 0.3	18.3 ± 2.0	0.6 ± 0.1	27 ± 2
0.2Ni/3TEOA+CB	2.0 ± 0.4	17.1 ± 2.2	0.5 ± 0.2	25 ± 4
0.2Mn/4TEOA+CB	1.3 ± 0.2	18.9 ± 2.1	0.7 ± 0.2	27 ± 3
0.2Ni/4TEOA+CB	1.5 ± 0.2	18.0 ± 2.0	0.6 ± 0.1	23 ± 2
0.2Mn/5TEOA+CB	1.2 ± 0.3	20.2 ± 2.3	0.7 ± 0.3	25 ± 4
0.2Ni/5TEOA+CB	1.7 ± 0.4	21.2 ± 2.2	0.6 ± 0.2	21 ± 3
Rubber Compounds Filled with SiO_2_				
ZnO/MgO+SiO_2_	7.3 ± 0.3	24.3 ± 2.1	0.4 ± 0.3	98 ± 3
0.2Mn/3TEOA+SiO_2_	6.7 ± 0.4	22.5 ± 2.3	0.7 ± 0.4	96 ± 3
0.2Ni/3TEOA+SiO_2_	5.7 ± 0.3	23.4 ± 2.2	0.6 ± 0.3	98 ± 4
0.2Mn/4TEOA+SiO_2_	4.4 ± 0.4	16.3 ± 2.4	0.7 ± 0.5	99 ± 4
0.2Ni/4TEOA+SiO_2_	5.4 ± 0.5	22.5 ± 2.5	0.7 ± 0.4	89 ± 5
0.2Mn/5TEOA+SiO_2_	5.4 ± 0.3	19.6 ± 2.3	0.6 ± 0.3	89 ± 3
0.2Ni/5TEOA+SiO_2_	5.3 ± 0.4	20.6 ± 2.3	0.7 ± 0.4	89 ± 4

**Table 3:** Tensile mechanical properties of CR vulcanizates: SE_50_, SE_100_, SE_150_-stress at 50%, 100%, 150% relative elongation of the vulcanizate, TS-tensile strength, EB-elongation at break.

Vulcanizate	SE_50_ (MPa)	SE_100_ (MPa)	SE_150_ (MPa)	TS (MPa)	EB (%)
Vulcanizates Filled With CB					
ZnO/MgO+CB	2.7 ± 0.2	5.7 ± 0.2	10.2 ± 0.5	22 ± 0.9	288 ± 11
0.2Mn/3TEOA+CB	2.9 ± 0.3	7.5 ± 1.0	13.9 ± 0.7	20.6 ± 1.2	189 ± 9
0.2Ni/3TEOA+CB	3.2 ± 0.4	8.5 ± 1.0	15.0 ± 0.8	18.5 ± 0.6	167 ± 7
0.2Mn/4TEOA+CB	2.6 ± 0.3	7.1 ± 0.4	14.2 ± 0.9	16.2 ± 1.8	162 ± 12
0.2Ni/4TEOA+CB	2.4 ± 0.4	6.4 ± 0.7	12.4 ± 0.9	17.3 ± 0.7	176 ± 12
0.2Mn/5TEOA+CB	3.2 ± 0.2	7.8 ± 0.7	15.8 ± 0.6	17 ± 1.8	156 ± 11
0.2Ni/5TEOA+CB	3.5 ± 0.3	9.5 ± 0.4	-	16 ± 1.4	138 ± 9
Vulcanizates Filled With SiO_2_					
ZnO/MgO+SiO_2_	8.8 ± 0.5	-	-	13 ± 1.0	91 ± 13
0.2Mn/3TEOA+SiO_2_	3.8 ± 0.4	7.2 ± 0.3	9.4 ± 0.5	11.8 ± 0.6	198 ± 16
0.2Ni/3TEOA+SiO_2_	3.4 ± 0.5	5.7 ± 0.7	7.5 ± 0.6	10.3 ± 0.4	234 ± 20
0.2Mn/4TEOA+SiO_2_	4.3 ± 0.3	6.4 ± 0.3	8.5 ± 0.4	12.7 ± 0.8	302 ± 38
0.2Ni/4TEOA+SiO_2_	4.9 ± 0.7	8.8 ± 2.3	10.9 ± 1.1	12.4 ± 0.6	198 ± 56
0.2Mn/5TEOA+SiO_2_	3.2 ± 0.3	4.1 ± 0.4	5.1 ± 0.4	11.4 ± 1.9	325 ± 40
0.2Ni/5TEOA+SiO_2_	5.8 ± 0.4	8.5 ± 0.6	10.1 ± 0.6	13.6 ± 0.4	220 ± 19

**Table 4:** Hardness and aging factor of CR vulcanizates: A_f_-aging coefficient, H-Shore A hardness.

Vulcanizate	A_f_ (-)	H (ShA)
Vulcanizates Filled with CB		
ZnO/MgO+CB	1.0 ± 0.01	68 ± 1.7
0.2Mn/3TEOA+CB	0.6 ± 0.02	59 ± 0.4
0.2Ni/3TEOA+CB	0.8 ± 0.01	61 ± 0.5
0.2Mn/4TEOA+CB	0.6 ± 0.03	60 ± 0.4
0.2Ni/4TEOA+CB	0.6 ± 0.02	59 ± 0.9
0.2Mn/5TEOA+CB	0.6 ± 0.01	63 ± 0.3
0.2Ni/5TEOA+CB	0.5 ± 0.02	63 ± 0.4
Vulcanizates Filled with SiO_2_		
ZnO/MgO+SiO_2_	0.3 ± 0.02	84 ± 0.7
0.2Mn/3TEOA+SiO_2_	0.6 ± 0.02	71 ± 1.5
0.2Ni/3TEOA+SiO_2_	0.4 ± 0.03	76 ± 1.6
0.2Mn/4TEOA+SiO_2_	0.2 ± 0.04	74 ± 0.8
0.2Ni/4TEOA+SiO_2_	0.3 ± 0.07	79 ± 0.75
0.2Mn/5TEOA+SiO_2_	0.3 ± 0.05	75 ± 0.9
0.2Ni/5TEOA+SiO_2_	0.3 ± 0.03	75 ± 0.7

## Data Availability

The data presented in this study are available on request from the corresponding author.
